# Adverse Social Exposome During the Life Course and Vascular Brain Injury

**DOI:** 10.1001/jamanetworkopen.2025.12289

**Published:** 2025-05-27

**Authors:** Sarah A. Keller, Amanda DeWitt, W. Ryan Powell, Frederick B. Ketchum, Robert A. Rissman, Barbara B. Bendlin, Amy J. H. Kind

**Affiliations:** 1 Center for Health Disparities Research, University of Wisconsin School of Medicine and Public Health, Madison; 2 Department of Medicine, Geriatrics Division, University of Wisconsin School of Medicine and Public Health, Madison; 3 Department of Neurology, University of Wisconsin School of Medicine and Public Health, Madison; 4 Department of Physiology and Neuroscience and Alzheimer’s Therapeutic Research Institute, Keck School of Medicine of USC, San Diego, California; 5 Wisconsin Alzheimer’s Disease Research Center, University of Wisconsin School of Medicine and Public Health, Madison

## Abstract

**Importance:**

The adverse social exposome, the entirety of harmful lifetime social-environmental exposures, is associated with Alzheimer disease and related dementias (ADRD) risk and may be key to informing modifiable systemic and behavioral risk interventions. With increasing emphasis to understand lifetime risk, methods are needed to measure and study social exposome relative to ADRD-related biological processes, including cerebrovascular disease.

**Objective:**

To evaluate the association between adverse social exposome and the burden of vascular brain injury (VBI) post mortem.

**Design, Setting, and Participants:**

This retrospective cohort study analyzed brain bank donors from 2 Alzheimer Disease Research Centers. Donations occurred between January 5, 2000, and August 5, 2018. Analysis was performed between January 13 and July 20, 2023.

**Exposures:**

Adverse social exposome, via geo-linked, time-concordant Area Deprivation Index (ADI) rankings for each year of a donor’s lifetime.

**Main Outcomes and Measures:**

The main outcome consisted of the presence or absence of VBI indicators: infarcts, microinfarcts, hemorrhages, and white matter rarefaction. Association between cumulative or change in life-course ADI and VBI burden was evaluated using multivariate logistic regression models adjusted for sex and age at death.

**Results:**

The sample included 740 donors (417 female [56.4%]; mean [SD] age at death, 81.5 [10.3] years; mean [SD] VBI score, 0.7 [1.0]; and median life course ADI, 7.08 [IQR, 4.33-9.83]), with residential years spanning 1905 to 2018. In adjusted models, living more years in a county with an ADI ranked above the study population median was associated with increased VBI odds (adjusted odds ratio [AOR], 1.04; 95% CI, 1.03-1.05). Greater odds of VBI were identified in those with increasing ADI between youth and adulthood (AOR, 3.67; 95% CI, 3.65-3.70) and youth and older adulthood (AOR, 12.02; 95% CI, 7.87-18.35). Increased odds were found in groups with stable high median ADI from youth to adulthood (AOR, 3.08; 95% CI, 3.01-3.14) and youth to older adulthood (AOR, 8.46; 95% CI, 8.04-8.90).

**Conclusions and Relevance:**

In this cohort study of 740 brain bank donors, an association between ADI and the presence of VBI was indicated, when considering the full life course as well as specific life course periods. These results show promise for identifying geographic areas in need of targeted interventions to reduce cerebrovascular disease and ADRD risk factors.

## Key Points

**Question:**

What is the association between social and environmental exposures during the life course, measured by the Area Deprivation Index, and small markers of cerebrovascular disease known as vascular brain injury (VBI) found in a sample of brain bank donors?

**Findings:**

In this cohort study of 740 donors, odds of any VBI increased by about 4% with each year spent in a higher than median disadvantaged context.

**Meaning:**

These findings suggest an association between exposure to social and environmental disadvantage during the life course and the postmortem presence of VBI.

## Teaser

This cohort study evaluates the association of the postmortem burden of vascular brain injury with cumulative measures of and changes in exposure to harmful social-environmental factors during the life course among brain bank donors.

## Introduction

Cerebrovascular disease disproportionately affects individuals who experience socioeconomic disadvantage through disease processes such as hemorrhage and stroke.^1,2,3^ In the US, strokes are responsible for 1 of every 19 deaths and are the leading cause of disability.^4^ Cerebrovascular disease, particularly in small vessels, has been linked to impaired cognition^5^ as well as the development of vascular dementia, Alzheimer disease dementia, and other dementias.^6^ It is well established that brain health in adulthood and late life can be impacted by the social-environmental circumstances occurring during the life course, with 1 global study finding that 90% of population-attributable risk of stroke is associated with 10 modifiable systemic and behavioral risk factors.^7^ This concept known as the social exposome encompasses the complex ways socioeconomic circumstances, geographic context, culture, and institutions can influence health and create inequity above and beyond individual level factors.^8,9^ Investigators have identified relationships between cross-sectional measures occurring at a particular time in the social exposome and cerebrovascular disease, but a potential mechanism underlying these associations has not been identified, and more detailed exposure across the entire life course with linkage to biospecimens has yet to be assessed.

To help reduce risk of disease, it is critical to identify how the social exposome is associated with biological mechanisms responsible for cerebrovascular disease. Evidence shows that inequality created by social systems can interact with biology in a cumulative manner to result in disease over time.^10^ Additionally, the critical periods of development, or latency model, purport that exposures during certain developmental periods can have lifelong effects on health.^11^ The timing of these periods depends on the biological systems and outcomes of interest. However, there is a present gap in the literature examining the social exposome across the entire life course and the association between its cumulative effect and the timing of exposures with cerebrovascular disease identified in brain tissue.

The Area Deprivation Index (ADI) is a validated measure of the social exposome at a particular time. High ADI, or high exposure to adverse social exposome, has been widely associated with worse health outcomes, including hospital readmissions,^12^ post–ovarian cancer mortality,^13^ and increased rates of hypertension, diabetes, and obesity.^14^ Previous investigations have also identified cross-sectional links between high ADI and changes in brain structure and neurodegeneration.^15,16,17^ Researchers have found an association between higher ADI and increased risk for the presence of Alzheimer disease neuropathology in a cohort of brain bank donors.^15^ Additionally, existing longitudinal cohort studies have identified links between ADI and cerebral and hippocampal volume^16^ and between cognitive decline and cortical change.^17^ As of yet, no studies, to our knowledge, have summarized complete life course social exposome using the ADI.

Biobank tissue samples have been discussed as prime targets for exposomal research because they can be linked to area-level exposures using participant addresses and may already be linked to health outcome data.^18^ In the case of cerebrovascular disease, identification of lesions in tissue post mortem is the standard of validating a particular diagnosis when compared with imaging or clinical studies.^19^ Melcher et al^20^ previously published a method to link individual residential histories of brain donors to time-concordant ADI. Through the creation of residential history timelines and standard measures of cerebrovascular disease, it becomes possible to determine the association of life course social exposome with cerebrovascular disease. The present study is the first, to our knowledge, to use life course ADIs and aimed to evaluate the association between the postmortem burden of vascular brain injury (VBI) and both cumulative measures of the life course social exposome and change in social exposome over life course periods, as measured by the ADI. Determining the extent to which the lifetime exposure to high ADI is associated with VBI could provide additional evidence to establish its mechanistic underpinnings.

## Methods

### Study Sample

This cohort study is a retrospective analysis of brain donors from 2 Alzheimer Disease Research Center (ADRC) brain banks. We assessed 1253 donors for eligibility, with 513 ultimately excluded due to missing assessments of the outcome variables. Donations occurred between January 5, 2000, and August 5, 2018. Included donors had a birth and death date in their autopsy records, which is necessary for constructing address timelines, and had documented VBI lesion assessments (N = 740). A comparison of demographic characteristics across those included and excluded from analyses is provided in the eTable in Supplement 1. Informed consent was not required, as this study was deemed to not be human participant research by the Institutional Review Boards at the University of Wisconsin, Madison, and the University of California, San Diego. The Strengthening the Reporting of Observational Studies in Epidemiology (STROBE) reporting guideline for cohort studies was followed to ensure quality in reporting.

### Social Exposome Determination

The social exposome of this sample was determined by the ADI, a validated measure of 17 area-level indicators sourced from the US Census and American Community Survey. The University of Wisconsin Center for Health Disparities Research constructed 13 total discrete versions of the ADI for each decade between 1900 and the present, updated with historic census and geographic data to reflect changing area-level characteristics over time. Variables pertaining to education, employment, income, poverty, and housing characteristics are captured for each decade when available to ensure that each ADI is based on a similar framework of social determinants of health across decades. County-level ADIs are used across decades due to US Census data collection patterns and availability.

To determine ADIs across the life course for individual brain donors, we retrospectively determined residential histories using publicly available records.^20^ Trained archivists used the name, birth and death dates, last known address, and next of kin for each donor and identified public records matching known participant data. Public records used in timeline construction include, but are not limited to, birth certificates, marriage announcements, military records, and obituaries. A detailed report of the residential history construction methodology has been published elsewhere.^20^ Each address in the timeline is geographically linked to county-level, time-concordant ADI using geography information system software (ArcGIS, version 10.7; ESRI) and historical geocoding methods. The eMethods in Supplement 1 provides a discussion of missing ADIs in the residential history timelines.

**Figure 1.**
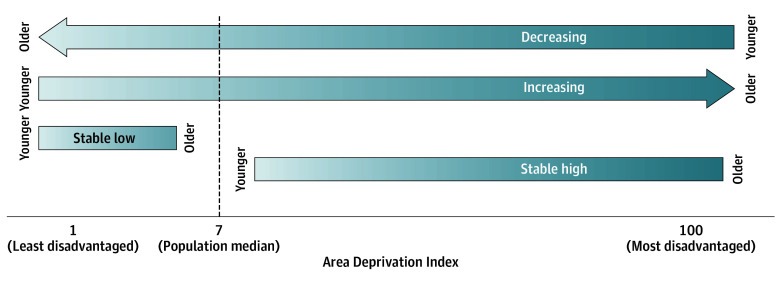
Change in Area Deprivation Index Categories

ADI values used ranged from 1 (least disadvantaged counties) to 100 (most disadvantaged counties). To capture a cumulative measure of the social exposome, the ADI was represented as the number of years spent living in a county with an ADI ranking above the median for the study population life course. This was chosen to improve power to estimate due to the relative homogeneity of ADI values across the life course in the sample. To capture the change in ADI over time, the life course of the group of donors was divided into 3 periods: youth (aged 0-18 years), adulthood (aged 19-64 years), and older adulthood (aged ≥65 years). These life periods were chosen based on the literature,^17^ the US legal adult age (18 years), and the typical US retirement age (65 years). Categorical indicators were created to represent the change in ADI between youth and adulthood and between youth and older adulthood stages. “Stable low” indicates an ADI less than or equal to the population median in both periods; “increasing” indicates an ADI less than or equal to the population median in the youth period and greater than the median in the older period; “decreasing” indicates an ADI greater than the median in the youth period and less than or equal to the median in the older period; and “stable high” indicates that the ADI was greater than the median in both periods (Figure 1).

### Brain Bank Neuropathological Data

Neuropathological data were drawn from National Alzheimer Coordinating Center (NACC) Neuropathology Data Form and autopsy records from the University of Wisconsin, Madison, and the University of California, San Diego, ADRC brain banks. As the Wisconsin ADRC was established most recently and only reported to NACC thereafter, brain bank data collected in years prior to the ADRC establishment were drawn from autopsy reports. For these records, a REDCap^21^ abstraction database was created to collect the relevant information by 3 trained abstractors (including S.A.K.) using standardized protocols and forms. All records were independently reabstracted by a blinded second abstractor, with strong interrater reliability and κ statistics ranging from 0.86 to 1.00 for all variables.

The VBI outcome measure is a score that combines lesions from the available autopsy data, first used by Godrich et al.^22^ The 4 included lesions are (1) infarcts, or small areas of tissue loss in the brain due to a lack of blood flow^23^; (2) microinfarcts, or similar areas of cell death that cannot be detected in gross assessments of pathology or magnetic resonance imaging^24^; (3) hemorrhages, or the accumulation of blood in the brain tissue^25^; and (4) white matter rarefaction, or changes to the white matter in the brain, including gliosis and loss of myelin and axons.^26^ The VBI outcome variable was coded as a dichotomized measure, either the absence of any VBI lesion (0) or the presence of any of the 4 possible lesions (1).

### Statistical Analysis

Analysis was performed between January 13 and July 20, 2023. Preliminary analyses generated descriptive statistics of key demographic variables, summaries of the change in ADI variables used to estimate outcomes, and the VBI outcomes. The life course social exposome was examined in 2 separate models as (1) a cumulative measure of the number of years spent above the median ADI of the population life course and (2) a measure of the change in ADI from youth to adulthood and older adulthood. The association between the life course measure of ADI and presence or absence of VBI lesions was modeled using multivariate logistic regression via SAS, version 9.4 (SAS Institute Inc), with the GENMOD procedure and log link function. Clustered SEs were included to account for systematic differences across the 2 brain bank locations. The models were also adjusted for age at death and sex. To ensure correct model specification, tests of the linearity of the quantitative age at death and ADI outcome variables were performed, and no evidence of nonlinearity was identified. No evidence of statistical interaction was identified after testing this between ADI and the covariates sex and age at death.

## Results

### Descriptive Statistics

The demographic composition of the sample is presented in Table 1. The analytic sample included data from 740 donors, with residential history years spanning 1905 to 2018. The mean (SD) age at death was 81.5 (10.3) years. A total of 417 donors (56.4%) were female and 323 (43.6%) were male. Six hundred and ninety-five donors (93.9%) lived to at least 65 years of age. In terms of race, 1 donor (0.1%) was identified as Asian; 3 (0.4%), Black or African American; 590 (79.7%), White; and 4 (0.5%), other (ie, no specific categories were provided). Thirty-six donors (4.9%) were identified as Hispanic or Latino ethnicity.

**Table 1.** Demographic Characteristics of Donor Sample

Characteristic	No. (%) of donors (N = 740)^a^
Age at death, mean (SD), y	81.5 (10.3)
Age group at death, y	
<65	45 (6.1)
65-69	64 (8.6)
70-74	67 (9.1)
75-79	87 (11.8)
80-84	140 (18.9)
85-89	168 (22.7)
≥90	169 (22.8)
Year of birth	
1900-1919	88 (11.9)
1920-1939	498 (67.3)
1940-1959	146 (19.7)
1960 and later	8 (1.1)
Year of death	
2000-2009	196 (26.5)
2010-2018	544 (73.5)
Sex	
Female	417 (56.4)
Male	323 (43.6)
Race	
Asian	1 (0.1)
Black or African American	3 (0.4)
White	590 (79.7)
Other^b^	4 (0.5)
No record of race available	142 (19.2)
Ethnicity	
Hispanic or Latino	36 (4.9)
Non-Hispanic	704 (95.1)
Marker of VBI^c^	
Infarcts	129 (17.4)
Microinfarcts	110 (14.9)
Hemorrhages	46 (6.2)
White matter rarefaction	255 (34.5)
Any VBI markers present	333 (45.0)
VBI score, mean (SD)^d^	0.7 (1.0)
Life course ADI, median (IQR)^e^	
Total	7.08 (4.33-9.83)
Youth (aged 0-18 y)	28.34 (6.34-50.34)
Adult (age 19-64 y)	7.09 (4.59-9.59)
Older adult (≥65 y)	7.06 (4.06-10.06)
No. of ADI life course years above the median, mean (SD)	19.95 (20.89)

Abbreviations: ADI, Area Deprivation Index; VBI, vascular brain injury.

^a^ Percentages have been rounded and may not equal 100.

^b^ Category is an artifact of the autopsy records, and thus no specific categories can be provided.

^c^ Percentages for markers of VBI represent the number of the sample for whom each marker was found on autopsy, so they will not equal 100.

^d^ Scores range from 0 (no evidence of VBI) to 4 (presence of all lesions).

^e^ Median ADIs calculated using all time-concordant ADI percentiles for each available year and filling in missing ADIs with linear interpolation.

### Indicators of VBI

The burden of the individual components and mean VBI score are presented in Table 1. The most common neuropathological lesion documented as present in the sample was white matter rarefaction (255 [34.5%]), while hemorrhages were the least common (46 [6.2%]). The mean (SD) VBI score was 0.7 (1.0), indicating mild burden across the sample. More than half of the sample (407 [55.0%]) had a VBI score of 0, denoting no documented evidence of VBI.

### Cumulative Life Course ADI and Association With Any VBI

**Figure 2.**
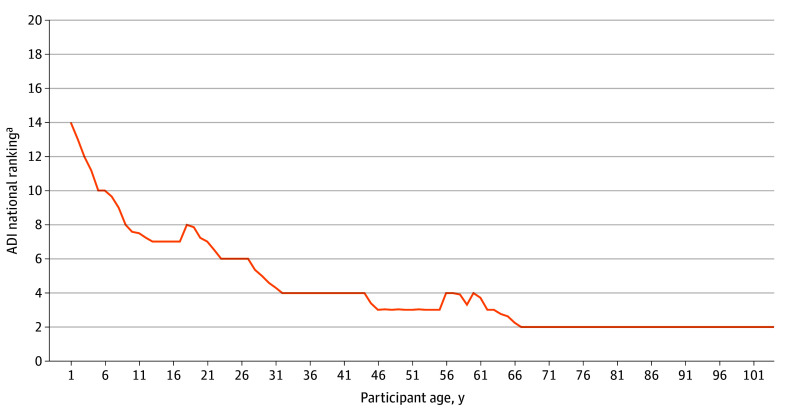
Median Life Course Area Deprivation Index (ADI) by Participant Age ^a^Greater ADI indicates greater disadvantage.

Figure 2 depicts the median ADI in the sample across the lifespan. The median life course ADI in this population was 7.08 (IQR, 4.33-9.83). Six hundred and fifty-one donors (88.0%) spent at least 1 year living in a county above the population median. The mean (SD) number of years during the life course above the median ADI was 19.95 (20.89; range, 0-90). While the median ADIs presented in Figure 2 remain relatively low over time, they do not capture the fluctuations in exposure to high ADI disadvantage that the brain bank donors experienced during their life courses. The eFigure in Supplement 1 illustrates the variation in ADI both within and between individuals in the sample over time. In the adjusted model, there was an estimated 4% increase in the odds of VBI for every additional year spent in a county with an ADI greater than the population median (adjusted odds ratio [AOR], 1.04; 95% CI, 1.03-1.05).

### Change in Life Course ADI and Any VBI

The median ADI for donors in their youth was 28.34 (IQR, 6.34-50.34), while the adult and older adult periods were exposed less overall, with medians of 7.09 (IQR, 4.59-9.59) and 7.06 (IQR, 4.06-10.06), respectively. Table 2 presents the change in ADI between the youth and adulthood periods and between the youth and older adulthood periods. In both the transition from youth to adulthood and from youth to older adulthood, the stable low category was most frequent (269 of 740 [36.4%] and 197 of 695 [28.3%], respectively), indicating an overall low exposure to social exposome disadvantage in the sample. The increasing category was the least frequently found in our sample, both in the change to adulthood (81 of 740 [10.9%]) and older adulthood (129 of 695 [18.6%]).

**Table 2.** Change in ADI From Youth

ADI change category^a^	Frequency, No. (%)
**Youth (aged 0-18 y) to adulthood (aged 19-64 y) (N = 740)**	
Stable low	269 (36.4)
Increasing	81 (10.9)
Decreasing	210 (28.38)
Stable high	180 (24.32)
**Youth (aged 0-18 y) to older adulthood (aged ≥65 y) (n = 695)**	
Stable low	197 (28.3)
Increasing	129 (18.6)
Decreasing	196 (28.20)
Stable high	173 (24.89)

Abbreviation: ADI, Area Deprivation Index.

^a^ Stable low indicates the ADI was less than or equal to the population median in both periods; increasing, an ADI less than or equal to the median in the youth period and greater than the median in the older period; decreasing, an ADI greater than the population median in the youth period and less than or equal to the median in the older period; and stable high, the ADI was greater than the median in both periods. Greater ADI indicates greater disadvantage.

Table 3 presents the odds of VBI associated with change in ADI by life stage. Increasing median ADI from youth to adulthood was associated with highest odds of VBI when compared with the stable low group (AOR, 3.67; 95% CI, 3.65-3.70). Stable high median ADIs from youth to adulthood were also associated with higher odds of VBI lesions (AOR, 3.08; 95% CI, 3.01-3.14). Similar results were returned from the analysis modeling the change between youth and older adulthood, with increased odds of VBI lesions in the increasing (AOR, 12.02; 95% CI, 7.87-18.35) and stable high (AOR, 8.46; 95% CI, 8.04-8.90) categories when compared with the stable low group.

**Table 3.** Unadjusted and Adjusted Odds of Vascular Brain Injury in Life Course ADI Groupings^a^

Characteristic	Odds ratio (95% CI)	
	Unadjusted	Adjusted^a^
**Change in ADI from youth (aged 0-18 y) to adulthood (aged 19-64 y)^b^**		
Stable low	1 [Reference]	1 [Reference]
Increasing	2.92 (1.74-4.88)	3.67 (3.65-3.70)
Decreasing	0.88 (0.60-1.28)	1.09 (0.95-1.25)
Stable high	2.83 (1.91-4.18)	3.08 (3.01-3.14)
**Change in ADI from youth (aged 0-18 y) to older adulthood (aged ≥65 y)^b^**		
Stable low	1 [Reference]	1 [Reference]
Increasing	8.02 (4.85-13.27)	12.02 (7.87-18.35)
Decreasing	0.80 (0.49-1.28)	0.82 (0.58-1.16)
Stable high	9.68 (6.01-15.58)	8.46 (8.04-8.90)

Abbreviation: ADI, Area Deprivation Index.

^a^ Adjusted for sex and age at death.

^b^ Stable low indicates the ADI was less than or equal to the population median in both periods; increasing, an ADI less than or equal to the median in the youth period and greater than the median in the older period; decreasing, an ADI greater than the population median in the youth period and less than or equal to the median in the older period; and stable high, the ADI was greater than the median in both periods. Greater ADI indicates greater disadvantage.

## Discussion

In this cohort study of 740 donors from 2 ADRC brain banks, exposure to high ADI at any point in the life course increased VBI risk. Odds of any VBI increased by about 4% with each year of above median ADI exposure. Increased odds of VBI lesions were also found in those with median ADIs that increase across the life course and in groups with stable ADIs higher than the population median across the life course. In this study, investigators were able to characterize the cumulative social exposome and changes in the social exposome over the life course, adding this information to well-phenotyped tissue within existing brain bank data. The novel residential history construction method^20^ allowed the study team to link time-concordant ADIs to 68.14% of person-years, which were summarized across the life course as both the number of years spent above the population median and comparisons across life course periods. This was the first time, to our knowledge, such social life course data has been added retrospectively to existing ADRC brain bank donors.

Previous reviews of the literature found that life-course exposome linkages to brain tissue are exceedingly rare,^27^ and longitudinal characterization of social exposome measure tend to exclude older adults.^28^ Including measurements of the social exposome, from birth to death, is crucial for determining how the dosage and timing of exposures can impact health outcomes. This work using objective measures of the life course social exposome linked to biobanked samples is the first step in exploring whether critical or sensitive periods of exposure to adverse social exposome may create or exacerbate disparities.

The present study builds on foundational work from existing longitudinal cohorts and studies of biobanked samples to understand the risk factors that contribute to the development of Alzheimer disease and related dementias (ADRD) pathology.^29,30,31^ However, gaps remain in determining the comprehensive life course social exposures necessary to better understand the link between the exposome and development of ADRD pathology. Moreover, studies are needed that increase the geographic diversity of brain bank samples. The present study only used 2 sites, but by leveraging a national sample of brain bank donors and life course ADIs, future investigators could open pathways to identify the causal mechanism between high ADI and ADRD pathology.

Future analyses could investigate how this association is mediated by risk factors already linked to brain health, including physical activity, hypertension, and smoking, to elucidate how living in high ADI contexts produces brain pathology. Other studies have found that modifiable risk factors significantly mediate the association between ADI and health outcomes,^16,32^ but this has yet to be examined using life course ADI summaries. Continuing to build this body of work can help curate targeted public health interventions addressing these modifiable risk factors in specific geographic areas and time periods in the life course where such intervention would have the greatest impact.

### Limitations

The present study has some limitations that should be considered along with these results. First, the sample is not representative of the broader US population. Previous work has established that those donating to biobanks tend to be less socioeconomically disadvantaged and overwhelmingly White,^33,34^ which is consistent with our findings. We also relied on autopsy records collected over several decades, which resulted in a large proportion of our sample with missing or unknown race and ethnicity data. These are known issues among ADRC brain banks, and efforts to make these biobanks more generalizable are currently under way.^35,36,37,38^ Improving the geographic, socioeconomic, and racial and ethnic diversity in brain banks is critical for furthering research in this area. Additionally, the neuropathological data that contributed to the VBI outcome variable was collected over decades, with autopsies occurring after the death of each participant. In this time, standards of measurement and the definitions of the relevant lesions may have changed, potentially impacting the results of the present study. However, the NACC has made a considerable effort to standardize data collection in affiliated brain banks to reduce variability over time.

## Conclusions

In this cohort study across 2 brain banks, the cumulative dose and timing of life course exposures of high ADI were associated with higher risk of VBI. Future studies should apply these methods to a donor sample more representative of patient populations in the US to help establish the mechanism driving the link between the adverse social exposome and cerebrovascular disease. This could allow clinicians and researchers to target specific geographic areas for intervention on modifiable risk factors across the life course.

## Notes

Supplement 1. **eTable.** Demographic Characteristics of Entire Decedent Sample **eFigure.** Person-Level ADI by Residential History Year **eMethods.** Missingness and Imputation of Area Deprivation Index (ADI) Values

Supplement 2.
Data Sharing Statement
