# Supplementary material for: Adverse Social Exposome During the Life Course and Vascular Brain Injury
Source: JAMA Netw Open. 2025 May 27;8(5):e2512289. doi: 10.1001/jamanetworkopen.2025.12289 (PMC12117465; doi:10.1001/jamanetworkopen.2025.12289)
Supplement: Supplement 1. — eTable. Demographic Characteristics of Entire Decedent Sample eFigure. Person-Level ADI by Residential History Year eMethods. Missingness and Imputation of Area Deprivation Index (ADI) Values [file jamanetwopen-e2512289-s001.pdf]

Supplementary Online Content

Keller SA, DeWitt A, Powell WR, et al. The adverse social exposome during the life course and vascular brain injury. *JAMA Netw Open.* 2025;8(5):e2512289. doi:10.1001/jamanetworkopen.2025.12289

eTable. Demographic Characteristics of Entire Decedent Sample

eFigure. Person-Level ADI by Residential History Year

eMethods. Missingness and Imputation of Area Deprivation Index (ADI) Values

This supplementary material has been provided by the authors to give readers additional information about their work.

© 2025 Keller SA et al. *JAMA Network Open*.

eTable. Demographic Characteristics of Entire Decedent Sample

Characteristic	Total Sample N=1253	Sample Not Missing Vascular Variables n=740	Sample Missing Vascular Variables n=513
Age at Death, Mean (SD)	80.9 (10.0)	81.5 (10.3)	80.1 (9.31)
Age Group at Death, no. (%)			
<65	67 (5.35)	45 (6.08)	22 (4.29)
65-69	103 (8.22)	64 (8.65)	39 (7.60)
70-74	127 (10.14)	67 (9.05)	60 (11.70)
75-79	195 (15.56)	87 (11.76)	108 (21.05)
80-84	262 (20.90)	140 (18.92)	122 (23.78)
85-89	260(20.75)	168 (22.70)	92 (17.93)
≥ 90	239 (19.07)	169 (22.84)	70 (13.65)
Year of Birth, no. (%)			
Before 1900	9 (0.72)	0 (0)	9 (1.75)
1900-1909	84 (6.70)	3 (0.41)	81 (15.79)
1910-1919	302 (24.10)	85 (11.49)	217 (42.30)
1920-1929	460 (36.71)	308 (41.62)	152 (29.63)
1930-1939	231 (18.44)	190 (25.68)	41(7.99)
1940-1949	119 (9.50)	111 (15.00)	8 (1.56)
1950-1959	39 (3.11)	35 (4.73)	4 (0.78)
1960 and later	9 (0.72)	8 (1.08)	1 (0.19)
Year of Death, no. (%)			
1980-1989	51 (4.07)	0 (0)	51 (9.94)
1990-1999	250 (19.95)	0 (0)	250 (48.73)
2000-2009	398 (31.76)	196 (26.49)	202 (39.38)
2010-2018	554 (44.21)	544 (73.51)	10 (1.95)
Sex, no. (%)			
Female	418 (55.96)	417 (56.35)	1 (0.20)
Male	329 (44.04)	323 (43.65)	6 (1.17)
Race, no. (%)			

© 2025 Keller SA et al. *JAMA Network Open*.

	White	1063 (84.84)	590 (79.73)	473 (92.20)
	Hispanic or Latino	37 (2.95)	36 (7.81)	1 (0.19)
	Black or African American	9 (0.72)	3 (0.41)	6 (1.17)
	Asian	2 (0.16)	1 (0.14)	1 (0.19)
	Other	7 (0.56)	4 (0.54)	3 (0.59)
	More than one race	3 (0.24)	0 (0)	3 (0.58)
	No record of race available	169 (13.49)	142 (19.19)	27 (5.26)

© 2025 Keller SA et al. *JAMA Network Open*.

eFigure. Person-Level[a] ADI by Subject Age

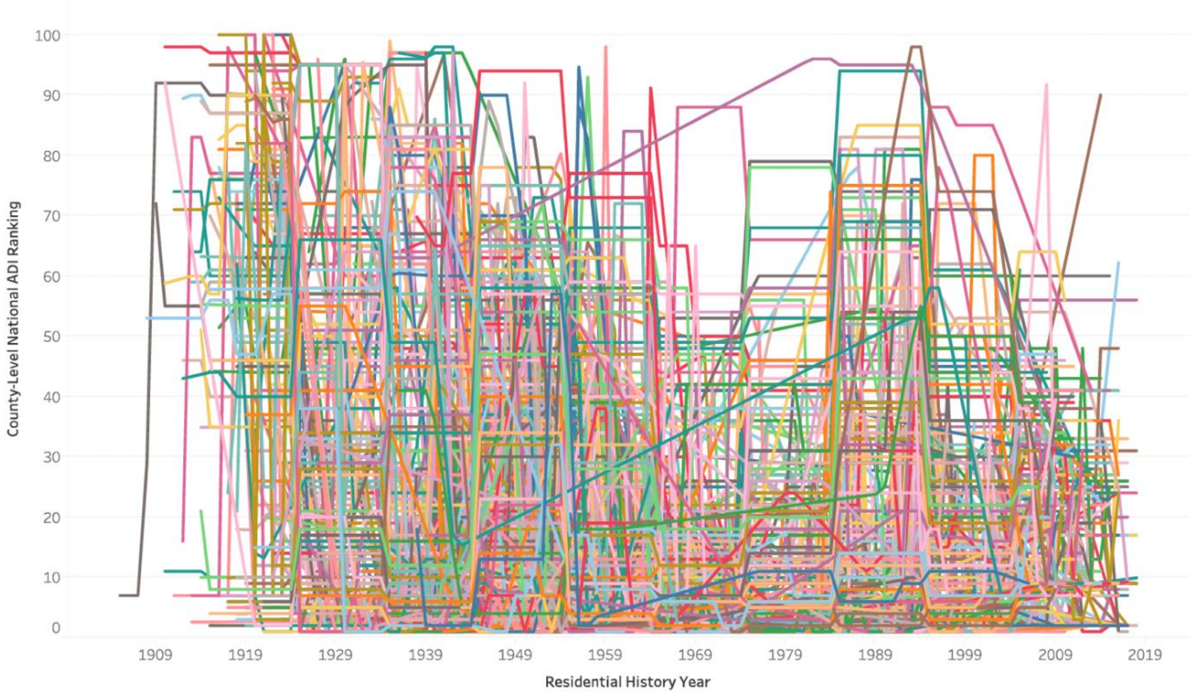

[a] Each line represents an individual donor's ADI across their life course

© 2025 Keller SA et al. *JAMA Network Open*.

eMethods. Missingness and Imputation of Area Deprivation Index (ADI) Values

Among the 740 decedents in the final sample, some addresses were unable to be geocoded and linked to a time-concordant ADI percentiles. 34460 of 108232 total person years were missing ADI values (31.84%). Geocoding and historical ADI linkage is an ongoing process, so the degree of missingness will be improved in future analyses of this sample. To address this missingness, we used linear interpolation, assuming a linear progression of ADI between two non-missing points.

© 2025 Keller SA et al. *JAMA Network Open*.
