# Supplementary material for: Adverse Social Exposome During the Life Course and Vascular Brain Injury
Source: JAMA Netw Open. 2025 May 27;8(5):e2512289. doi: 10.1001/jamanetworkopen.2025.12289 (PMC12117465; doi:10.1001/jamanetworkopen.2025.12289)
Supplement: Supplement 2. — Data Sharing Statement [file jamanetwopen-e2512289-s002.pdf]

Data Sharing Statement

Keller. Adverse Social Exposome During the Life Course and Vascular Brain Injury. *JAMA Netw Open*. Published May 27, 2025. doi:10.1001/jamanetworkopen.2025.12289

Data
Data available: No

Additional Information
Explanation for why data not available: This manuscript represents a subset of a larger, ongoing study and so the data cannot be made available at this time
